# Auryon 355 nm Laser Atherectomy for Femoropopliteal In-Stent Occlusions: A Retrospective Comparative Study of Technical Success and 12-Month Outcomes

**DOI:** 10.3390/biomedicines14071538

**Published:** 2026-07-09

**Authors:** Antonio Marzano, Federico Flora, Olga Silvestri, Maria Stella De Chiara, Danilo Barbarisi, Vito Gallicchio, Loris Flora, Luca di Marzo, Antonio Peluso, Wassim Mansour

**Affiliations:** 1 Vascular Surgery Unit, Department of General Surgery , Surgical Specialties and Anesthesiology, “Sapienza” University of Rome, 00161 Rome, Italy; luca.dimarzo@uniroma1.it (L.d.M.); wassim.mansour@uniroma1.it (W.M.); 2 Vascular Surgery Unit, Heart and Vascular Department, A.O.R.N. “San Giuseppe Moscati”, 83100 Avellino, Italy; federico.flora@virgilio.it (F.F.); olga.silvestri@unina.it (O.S.); mstella.dechiara@libero.it (M.S.D.C.); danilo.barbarisi@gmail.com (D.B.); vitogallicchio@gmail.com (V.G.); loflora7@gmail.com (L.F.); antoniopeluso8784@gmail.com (A.P.); 3 Department of Public Health, University of Naples “Federico II”, 80131 Naples, Italy

**Keywords:** peripheral arterial disease, PAD, femoropopliteal disease, in-stent occlusion, in-stent restenosis, laser atherectomy, Auryon, endovascular recanalization, target-lesion revascularization

## Abstract

**Background**: Complete femoropopliteal in-stent occlusions remain technically challenging because durable treatment requires restoration of an intraluminal channel within the pre-existing stent scaffold. This study evaluated Auryon 355 nm laser-assisted recanalization compared with conventional endovascular recanalization in symptomatic patients with femoropopliteal in-stent occlusions. **Methods**: This retrospective single-center comparative study included patients treated with Auryon laser-assisted recanalization between May 2023 and June 2024 and a historical control group treated with conventional recanalization between January 2020 and April 2022. The prespecified primary endpoint was a conditional endpoint of 12-month primary patency among primary-strategy technically successful procedures. An exploratory full-cohort strategy-level analysis of 12-month durable technical success was also performed to account for initial technical failures. **Results**: Forty-four patients were analyzed: 18 in the Auryon group and 26 in the control group. Baseline clinical characteristics were broadly comparable, although selected anatomical and clinical imbalances were present. Post hoc sensitivity analyses were considered hypothesis-generating because of sparse events and wide confidence intervals. Primary-strategy technical success was significantly higher with Auryon-assisted recanalization than with conventional recanalization (94.4% vs. 65.4%, *p* = 0.031). In the conventional group, six of nine primary-strategy failures were subsequently recanalized after distal retrograde bailout puncture, yielding a descriptive final assisted recanalization rate of 88.5% (23/26). In the full-cohort strategy-level analysis, 12-month durable technical success was achieved in 83.3% of patients in the Auryon group and 50.0% in the control group (*p* = 0.030). Among primary-strategy technically successful procedures, 12-month primary patency was numerically higher after Auryon-assisted treatment (88.2% vs. 76.5%; log-rank *p* = 0.37), without statistically significant differences in patency-related secondary outcomes. No distal embolization or procedure-related complication occurred in the Auryon group. **Conclusions**: In this retrospective comparative experience, Auryon 355 nm laser atherectomy was associated with significantly higher primary-strategy technical success than conventional endovascular recanalization. Among successfully recanalized patients, 12-month patency-related outcomes were numerically favorable but not statistically significant. Larger prospective studies are warranted.

## 1. Introduction

Peripheral arterial disease (PAD) is a common manifestation of systemic atherosclerosis and may progress from intermittent claudication to chronic limb-threatening ischemia (CLTI) [1,2,3]. Endovascular therapy represents a central treatment option for infrainguinal disease, particularly in patients at increased surgical risk or with anatomy suitable for percutaneous revascularization [2,4,5]. In the femoropopliteal segment, balloon angioplasty, drug-coated balloons (DCBs), bare-metal stents, drug-eluting stents, and covered stents have all been used to improve acute technical success and mid-term patency [6,7,8,9,10]. However, restenosis and occlusion after femoropopliteal stenting remain clinically relevant limitations.

Laser atherectomy has been proposed as a vessel-preparation strategy for complex femoropopliteal in-stent restenosis and occlusion, with the aim of modifying neointimal or thrombotic material within the pre-existing stent scaffold before adjunctive balloon angioplasty [11,12]. The Auryon laser atherectomy system (AngioDynamics, Inc., Latham, NY, USA) is a solid-state 355 nm laser platform used for peripheral atherectomy. Preclinical and early clinical studies have suggested that this wavelength may allow plaque modification with limited thermal injury, although the clinical relevance of these mechanistic findings requires further evaluation [13,14,15,16,17]. In the present study, the 2.0 mm and 2.35 mm catheters were used according to vessel diameter, stent characteristics, access profile, and operator judgment.

Early prospective and real-world studies evaluating the 355 nm laser platform reported favorable acute safety and technical performance, with encouraging outcomes at 6 and 12 months in heterogeneous populations with infrainguinal PAD [18,19,20]. However, these investigations included mixed lesion subsets, and focused comparative evidence regarding complete femoropopliteal in-stent occlusions remains limited. Furthermore, device-based revascularization should always be integrated into a comprehensive secondary prevention strategy, as high-intensity statin therapy has been associated with improved survival in patients with PAD [21].

Accordingly, this study aimed to evaluate the short-term safety and effectiveness of Auryon laser-assisted endovascular recanalization compared with conventional endovascular recanalization in symptomatic patients with femoropopliteal in-stent occlusions.

## 2. Materials and Methods

### 2.1. Study Design

This was a retrospective, single-center, comparative cohort study conducted at a vascular surgery unit with experience in endovascular treatment of lower-limb arterial disease. Consecutive patients with symptomatic PAD and femoropopliteal in-stent occlusions treated with Auryon laser-assisted endovascular recanalization between May 2023 and June 2024 were analyzed and compared with a historical control group of patients treated with conventional endovascular recanalization between January 2020 and April 2022.

The same operator team performed procedures during both study periods, and the indications for treatment, eligibility criteria, adjunctive lesion-treatment protocol after successful recanalization, postprocedural antithrombotic therapy, and follow-up schedule remained unchanged over time. The interval between April 2022 and May 2023 was not included because Auryon-assisted recanalization had not yet been routinely implemented for femoropopliteal in-stent occlusions at our institution, and this transition period was excluded to preserve a clear comparison between conventional recanalization and the subsequent standardized Auryon-assisted strategy.

The study was designed and reported according to the principles of observational cohort research. Because of the retrospective nature of the analysis, all data were extracted from prospectively maintained institutional records, procedural reports, imaging archives, and follow-up files.

### 2.2. Study Population

All consecutive patients with symptomatic peripheral arterial disease and femoropopliteal in-stent occlusion treated during the predefined study periods were screened for eligibility. Patients were allocated to the Auryon group or the control group according to the endovascular recanalization strategy received. Eligibility was assessed using predefined clinical and anatomical inclusion and exclusion criteria.

Inclusion criteria were symptomatic PAD classified as Rutherford category 3, 4, or 5; femoropopliteal in-stent occlusion longer than 40 mm; at least one patent infragenicular runoff vessel with flow to the foot; and an estimated life expectancy longer than 1 year.

Exclusion criteria were Rutherford category 0, 1, 2, or 6; total occlusion length of the target femoropopliteal lesion, including the superficial femoral artery with or without popliteal extension, greater than 25 cm; poor distal runoff, defined as partial or complete occlusion of all three tibial vessels; multilevel atherosclerotic disease requiring additional revascularization procedures, such as common femoral endarterectomy or iliac stenting; and pre-planned major amputation.

The complete inclusion and exclusion criteria are summarized in Table 1.

### 2.3. Preoperative Assessment

All patients underwent preoperative clinical evaluation and computed tomography angiography for procedural planning. Demographic and clinical variables included age, sex, body mass index, smoking status, hypertension, diabetes mellitus, insulin-dependent diabetes, dyslipidemia, coronary artery disease, previous myocardial infarction, chronic kidney disease, previous amputation, and Rutherford category.

Anatomic and lesion-related variables included reference vessel diameter, total occlusion length, length of the in-stent occlusion, popliteal artery involvement, number of patent infragenicular runoff vessels, and access strategy. Lesion measurements were obtained from diagnostic angiography and computed tomography angiography using dedicated imaging software.

### 2.4. Endovascular Technique

All procedures were performed in an endovascular suite by experienced operators. Percutaneous arterial access was obtained using the Seldinger technique under ultrasound guidance. Ipsilateral antegrade common femoral access was preferred. Contralateral crossover access was used in cases of occlusion at the origin of the superficial femoral artery or when ipsilateral sheath positioning was not considered feasible.

In the Auryon group, recanalization was performed using a combined guidewire- and laser-assisted intraluminal technique. An initial attempt was made to advance a 0.014-inch guidewire through the occluded stent lumen. When complete guidewire traversal could not be achieved or when the supporting catheter could not be advanced through the occlusive material, the Auryon laser catheter was positioned at the proximal occlusive cap and activated to create a pilot channel before complete guidewire crossing. Short, controlled laser advancement was then alternated with guidewire progression, facilitating stepwise intraluminal recanalization of the occluded stent.

When the guidewire had already crossed the entire occlusion, the laser catheter was advanced over the wire and used primarily for modification and debulking of the intrastent occlusive material. Thus, Auryon was used both as an adjunctive crossing tool and as a vessel-preparation device, according to the technical characteristics of each lesion.

The 2.0 mm or 2.35 mm Auryon catheter was selected according to the nominal diameter of the occluded femoropopliteal stent, the estimated reference vessel diameter, access profile, and operator judgment. Active aspiration, available with both catheter sizes, was used throughout all Auryon-assisted procedures. After successful intraluminal recanalization, adjunctive lesion treatment was standardized in both groups. Predilatation and luminal optimization were first performed using plain balloon angioplasty, followed by DCB angioplasty. This sequence was applied routinely in all successfully recanalized in-stent occlusions as a standardized vessel-preparation strategy before definitive DCB delivery, rather than being reserved for selected lesion morphologies. Stent relining was reserved for residual stenosis, significant recoil, or flow-limiting dissection and was performed using uncovered self-expanding nitinol stents.

**Figure 1:**
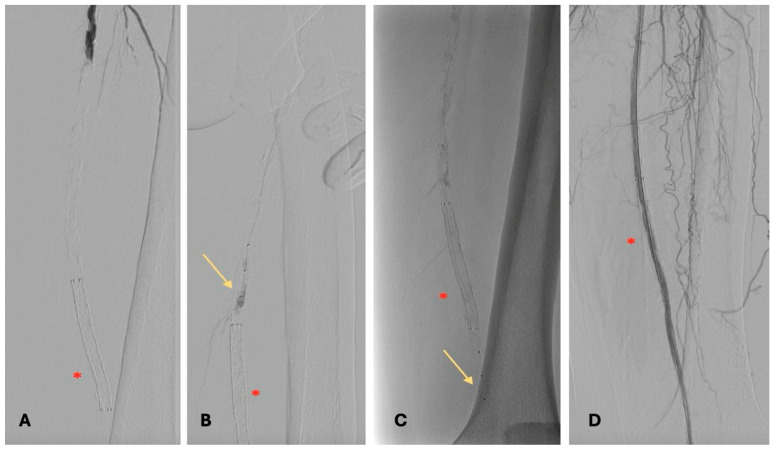
Representative angiographic case of Auryon-assisted recanalization of a femoropopliteal in-stent occlusion (*red asterisk: stent; yellow arrow: Auryon laser catheter*). (**A**) Baseline in-stent occlusion; (**B**) Auryon catheter within the occluded stent; (**C**) post-laser/balloon angioplasty; (**D**) final angiographic result.

A representative case of Auryon-assisted intraluminal recanalization of a femoropopliteal in-stent occlusion is shown in Figure 1.

In the control group, recanalization was performed using a stepwise conventional endovascular strategy. Crossing was attempted with hydrophilic 0.035-, 0.018-, and/or 0.014-inch guidewires supported by standard crossing and support catheters. Wire escalation and the selective knuckle-wire technique were used according to lesion morphology and operator judgment, provided that intrastent progression could be maintained within the pre-existing stent scaffold. When the occlusion could not be crossed from the planned access route, distal retrograde bailout puncture was attempted. After successful crossing, the same adjunctive lesion-treatment protocol used in the Auryon group was applied, consisting of plain balloon angioplasty followed by DCB angioplasty. Stent relining was used selectively in cases of an inadequate angiographic result, residual stenosis, recoil, or flow-limiting dissection and was performed using uncovered self-expanding nitinol stents.

Periprocedural anticoagulation and antiplatelet therapy were administered according to institutional practice. After technically successful recanalization, all patients in both groups received antithrombotic therapy according to the VOYAGER PAD regimen, consisting of acetylsalicylic acid 100 mg once daily plus rivaroxaban 2.5 mg twice daily [22,23].

### 2.5. Follow-Up Protocol

All included patients underwent scheduled clinical and duplex ultrasound follow-up at 30 days and at 3, 6, and 12 months after the index procedure. Additional clinical or imaging assessment was performed in the presence of recurrent or worsening symptoms, delayed wound healing, or suspected target-vessel re-occlusion.

Duplex ultrasound was used to assess target-vessel patency and detect restenosis or re-occlusion. Peak systolic velocity was measured within the treated femoropopliteal segment and compared with that in an adjacent reference segment. Hemodynamically significant restenosis was defined as a peak systolic velocity ratio >2.4. Target-vessel re-occlusion was defined as the absence of detectable flow within the treated femoropopliteal segment on duplex ultrasound. Confirmatory computed tomography angiography or catheter-based angiography was performed when clinically indicated.

### 2.6. Endpoints and Definitions

Primary-strategy technical success was defined as complete angiographic recanalization of the target arterial segment using the planned access strategy, restoration of inline flow, absence of flow-limiting dissection or major procedure-related complication, and residual stenosis ≤50% after balloon angioplasty alone or ≤30% after angioplasty with stent relining. The more permissive residual-stenosis threshold after balloon angioplasty alone reflected a pragmatic angiographic criterion for procedural success in the absence of recoil or flow-limiting dissection, whereas a stricter threshold was required after stent relining. Procedures requiring unplanned additional percutaneous access, such as distal retrograde puncture or direct stent puncture, were classified as primary-strategy technical failures, even when final vessel recanalization was subsequently achieved. Final assisted recanalization after bailout maneuvers was evaluated descriptively and was not part of the primary-strategy technical success definition.

The prespecified primary endpoint was a conditional endpoint of primary patency at 12 months among primary-strategy technically successful procedures. Primary patency was defined as freedom from target-vessel re-occlusion, hemodynamically significant restenosis on duplex ultrasound, identified by a peak systolic velocity ratio > 2.4, and CD-TLR. Loss of primary patency was defined by the first occurrence of any of these events. Target-vessel re-occlusion and CD-TLR were also evaluated separately as individual secondary endpoints.

Because primary patency could only be evaluated after successful target-vessel recanalization, it was analyzed conditionally among technically successful procedures. To provide an overall strategy-level estimate that also accounted for initial technical failures, an exploratory full-cohort composite endpoint of 12-month durable technical success was evaluated. Durable technical success was defined as successful index recanalization followed by maintenance of primary patency through 12 months. Patients with initial technical failure were classified as failures of this composite endpoint. An additional exploratory composite of technical success with freedom from CD-TLR through 12 months was also assessed in the full study cohort.

Secondary endpoints included primary-strategy technical success, final assisted recanalization after bailout maneuvers, clinically driven target-lesion revascularization (CD-TLR), major amputation of the treated limb, all-cause mortality, procedure-related complications, procedural time and length of postoperative hospital stay. CD-TLR was defined as repeat revascularization of the target lesion driven by recurrent symptoms, worsening ischemia, or objective evidence of target-vessel failure.

### 2.7. Statistical Analysis

Continuous variables were reported as mean ± standard deviation and compared using Welch’s two-sample *t*-test. Categorical variables were reported as counts and percentages and compared using the two-sample test for equality of proportions or Fisher’s exact test, as appropriate. Between-group differences were primarily reported as absolute risk differences with 95% confidence intervals, calculated as Auryon minus control. Standardized mean differences (SMDs) were calculated for baseline clinical, anatomical, and procedural variables to quantify between-group imbalance independently of sample size. Odds ratios, when reported, were oriented as Auryon versus control.

As a post hoc sensitivity analysis for technical success, Firth penalized logistic regression was performed because of the limited sample size and small number of technical failures. To avoid overfitting, separate parsimonious models were fitted including treatment group and one clinically relevant imbalance variable at a time, namely in-stent occlusion length, one-vessel runoff, or smoking history. These models were considered exploratory. A *p*-value < 0.05 was considered statistically significant. Statistical analyses were performed using R software, version 4.5.3 (R Foundation for Statistical Computing, Vienna, Austria), within RStudio Desktop, version 2026.05.0 (Posit Software, PBC, Boston, MA, USA).

Given the retrospective design and limited sample size, all comparative follow-up analyses were considered exploratory. Primary-strategy technical success was analyzed in the full study cohort. Final assisted recanalization after bailout maneuvers was summarized descriptively to distinguish absolute final technical failures from failures of the predefined primary recanalization strategy. Primary patency, target-vessel re-occlusion, and other patency-related outcomes were analyzed conditionally among technically successful procedures because a failed recanalization does not establish a patent treated segment eligible for subsequent patency assessment. Primary patency was analyzed using the Kaplan–Meier method, and between-group differences were assessed using the log-rank test, with loss of primary patency defined as the first occurrence of target-vessel re-occlusion, a duplex ultrasound peak systolic velocity ratio > 2.4, or CD-TLR. To address potential selection introduced by conditioning follow-up analyses on technical success, 12-month durable technical success and technical success with freedom from CD-TLR were additionally evaluated in the full cohort, with initial technical failures classified as composite endpoint failures. Absolute risk differences with 95% confidence intervals were calculated using the Newcombe method, and categorical comparisons were performed using Fisher’s exact test.

Procedural time and length of hospital stay were reported descriptively among patients with technically successful recanalization because corresponding data were not uniformly retrievable for patients with technical failure. Therefore, these conditional comparisons were not considered full-cohort estimates of procedural burden or resource use.

For non-significant 12-month outcomes, a post hoc power-context analysis was performed to estimate the minimum detectable absolute difference under the available sample size, using a two-sided alpha level of 0.05 and 80% power. This analysis was intended only to contextualize statistical imprecision and was not used for inferential decision-making.

## 3. Results

### 3.1. Study Population

**Figure 2:**
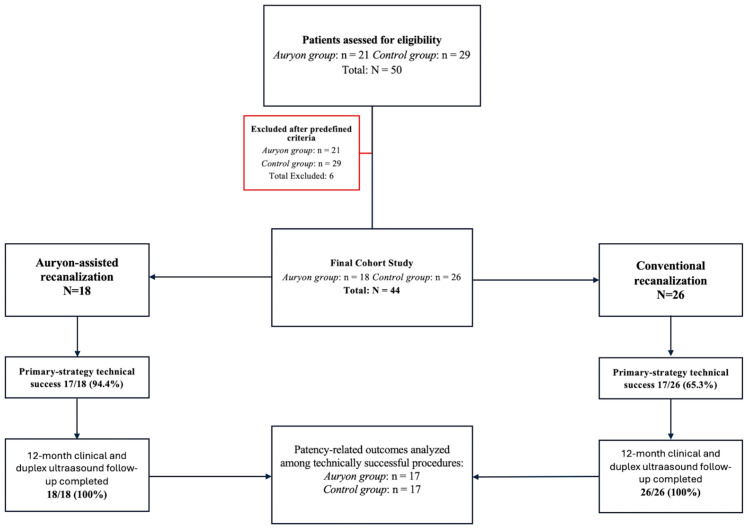
Study flow diagram.

Overall, 50 patients were assessed for eligibility: 21 patients treated with Auryon laser-assisted recanalization and 29 patients treated with conventional endovascular recanalization. After application of the predefined inclusion and exclusion criteria, six patients were excluded, three from each treatment group. Therefore, 44 patients were included in the final analysis: 18 in the Auryon group and 26 in the control group. The patient selection process is summarized in Figure 2.

The mean age of the overall cohort was 67.8 ± 9.3 years, and 30 patients were male (68.1%). No statistically significant baseline clinical differences were detected between groups. However, SMDs identified clinically relevant imbalances in selected variables, including smoking history, which was more frequent in the Auryon group, and chronic kidney disease, which was present only in the control group. Detailed baseline characteristics, including SMDs, are reported in Table 2. Overall, 22 patients (50.0%) were classified as Rutherford category 3, 17 (38.6%) as Rutherford category 4, and five (11.4%) as Rutherford category 5.

### 3.2. Lesion and Procedural Characteristics

Popliteal artery involvement was present in 13 patients (29.5%), without significant differences between groups. The mean reference vessel diameter was 5.2 ± 0.6 mm. The mean total occlusion length was 144 ± 50.3 mm and did not differ significantly between groups. However, in-stent occlusion length was significantly greater in the Auryon group than in the control group (97.2 ± 26.9 mm vs. 79.6 ± 19.4 mm, *p* = 0.024).

No statistically significant difference in infragenicular runoff was detected between groups; however, one-vessel runoff was numerically more frequent in the Auryon group and showed clinically relevant imbalance by SMD. Contralateral femoral crossover access was used in six patients (13.6%). Mean introducer size was significantly larger in the Auryon group than in the control group (6.7 ± 0.4 Fr vs. 5.8 ± 0.5 Fr, *p* < 0.001), consistent with the use of 2.0 mm and 2.35 mm laser catheters. Balloon diameter, balloon length, inflation pressure, number of balloons per patient, and need for stent relining did not differ significantly between groups. The adjunctive lesion-treatment strategy after successful recanalization did not differ between groups and consisted of plain balloon angioplasty followed by DCB angioplasty. When stent relining was required, uncovered self-expanding nitinol stents were used in both groups. Anatomical lesion characteristics and procedural variables are summarized in Table 3.

### 3.3. Primary-Strategy Technical Success and Final Assisted Recanalization

Primary-strategy technical success was achieved in 34 of 44 patients (77.3%). Primary-strategy technical success was significantly higher in the Auryon group than in the conventional recanalization group (17/18, 94.4% vs. 17/26, 65.4%; absolute difference, 29.1 percentage points; 95% CI, 3.8–48.8; *p* = 0.031). The single primary-strategy technical failure in the Auryon group was caused by inability to traverse the occlusion despite combined guidewire- and laser-assisted crossing attempts.

A case-by-case breakdown of all primary-strategy technical failures is provided in Supplementary Table S1. In the conventional group, nine primary-strategy technical failures occurred. Distal retrograde bailout puncture was attempted in all nine cases; final assisted recanalization was achieved in six cases, whereas three cases represented absolute final technical failures despite bailout. Accordingly, when bailout-assisted recanalization was considered descriptively, the final assisted recanalization rate in the conventional group was 23 of 26 procedures (88.5%). The single Auryon-group primary-strategy failure remained an absolute final technical failure despite combined guidewire-/laser-assisted crossing attempts and distal retrograde bailout puncture.

When expressed as an odds ratio oriented as Auryon versus control, Auryon-assisted recanalization was associated with higher odds of primary-strategy technical success in the unadjusted analysis (OR, 9.00; 95% CI, 1.02–79.4). In post hoc Firth penalized logistic regression sensitivity analyses, the direction of the treatment effect remained consistent after separate adjustment for in-stent occlusion length, one-vessel runoff, and smoking history. Because of the limited sample size, sparse number of failures, and wide confidence intervals, these post hoc models were considered hypothesis-generating sensitivity analyses and were not interpreted as adjusted evidence of a treatment effect. The results of the sensitivity analyses are reported in Supplementary Table S2.

### 3.4. Twelve-Month Outcomes

No patient was lost to follow-up. The scheduled 12-month clinical and duplex ultrasound assessment was available for all surviving patients; patients who experienced an event before 12 months were retained in the analysis and classified according to the first event observed. Postprocedural antithrombotic therapy was standardized across groups, with all patients receiving acetylsalicylic acid plus low-dose rivaroxaban according to the VOYAGER PAD regimen after recanalization.

Among technically successful procedures, primary patency at 12 months was maintained in 28 of 34 patients (82.4%): 15 of 17 patients in the Auryon group (88.2%) and 13 of 17 patients in the control group (76.5%). In this cohort, all losses of primary patency corresponded to target-vessel re-occlusion; no additional isolated hemodynamically significant restenosis or CD-TLR events occurred in otherwise patent target vessels. Accordingly, target-vessel re-occlusion occurred in six of 34 patients (17.6%), including two of 17 patients in the Auryon group (11.8%) and four of 17 patients in the control group (23.5%; *p* = 0.656).

CD-TLR occurred in five of 34 patients with technically successful recanalization (14.7%), with a numerically lower rate in the Auryon group than in the control group (1/17, 5.9% vs. 4/17, 23.5%; *p* = 0.335).

To account for the between-group difference in initial technical success, an exploratory strategy-level analysis was performed in the full study cohort. Primary-strategy durable technical success at 12 months, defined as successful index recanalization followed by maintenance of primary patency through 12 months, was achieved in 15 of 18 patients in the Auryon group and 13 of 26 patients in the control group (83.3% vs. 50.0%; *p* = 0.030). Accordingly, treatment-strategy failure, defined as either initial technical failure or subsequent target-vessel re-occlusion, occurred in three of 18 patients (16.7%) in the Auryon group and 13 of 26 patients (50.0%) in the control group. Primary-strategy technical success with freedom from CD-TLR through 12 months was achieved in 16 of 18 patients in the Auryon group and 13 of 26 patients in the control group (88.9% vs. 50.0%; *p* = 0.010). A post hoc power-context analysis for non-significant 12-month outcomes is reported in Supplementary Table S3. Given the available sample size, moderate differences in 12-month patency-related and clinical outcomes could not be reliably detected; therefore, non-significant *p*-values should not be interpreted as evidence of equivalence between groups.

In the full study cohort, major amputation occurred in three of 44 patients (6.8%): one of 18 patients in the Auryon group (5.6%) and two of 26 patients in the control group (7.7%). One death occurred during follow-up in the control group, corresponding to an overall mortality rate of 2.3% (0/18, 0% vs. 1/26, 3.8%). One procedure-related complication occurred in the control group (0/18, 0% vs. 1/26, 3.8%). No statistically significant between-group differences were observed in major amputation, all-cause mortality, or procedure-related complications. Despite the significantly larger introducer size used in the Auryon group, no major access-site complications were observed. Specifically, no pseudoaneurysm, arteriovenous fistula, access-related bleeding requiring transfusion or surgical/endovascular treatment, or clinically relevant groin hematoma requiring prolonged hospitalization occurred in the Auryon group.

The intraoperative complication observed in the control group consisted of distal embolization with subsequent occlusion of the peroneal artery after balloon predilation. The event remained clinically asymptomatic because of preserved patency of the tibial arteries and plantar arch. No distal embolization, arterial perforation, flow-limiting dissection, or other procedure-related complication was observed in the Auryon group.

Among primary-strategy technically successful procedures, mean procedural time was numerically longer in the Auryon group than in the control group (96.9 ± 45.1 vs. 75.2 ± 27.5 min; *p* = 0.080), although the difference was not statistically significant. Length of hospital stay was similar between groups (2.0 ± 1.8 vs. 2.1 ± 2.4 days; *p* = 0.857). Follow-up completeness is reported separately, and all 12-month outcomes were evaluated at the predefined 12-month time point. These comparisons were descriptive and conditional on technical success because corresponding data were not uniformly retrievable for patients with technical failure.

Technical success, exploratory strategy-level composite endpoints, full-cohort clinical outcomes, and conditional 12-month patency-related outcomes are reported in Table 4. Procedural time, length of hospital stay, and follow-up duration among technically successful procedures are summarized separately in Table 5 and should not be interpreted as full-cohort comparisons between treatment strategies.

**Figure 3:**
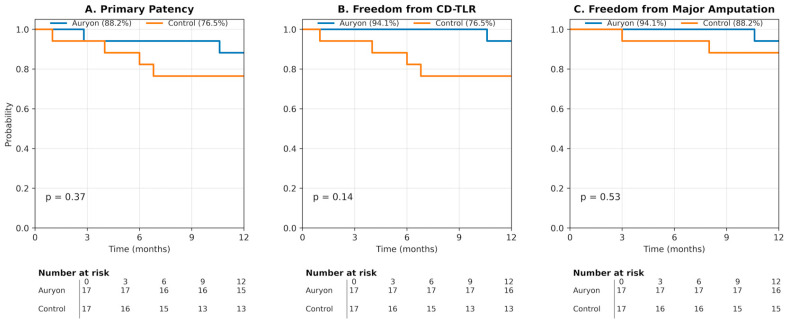
Kaplan–Meier estimates of 12-month outcomes after technically successful recanalization. (**A**) Primary patency; (**B**) freedom from clinically driven target-lesion revascularization (CD-TLR); (**C**) freedom from major amputation. The Auryon-assisted recanalization group was compared with the conventional endovascular recanalization group using the log-rank test. Numbers at risk are reported below each panel.

Kaplan–Meier analysis showed numerically higher 12-month primary patency in the Auryon group than in the control group (88.2% vs. 76.5%; log-rank *p* = 0.37). Freedom from CD-TLR at 12 months was 94.1% in the Auryon group and 76.5% in the control group (log-rank *p* = 0.14), whereas freedom from major amputation was 94.1% and 88.2%, respectively (log-rank *p* = 0.53). Kaplan–Meier estimates and the corresponding numbers at risk are shown in Figure 3.

## 4. Discussion

This retrospective comparative study evaluated Auryon 355 nm laser atherectomy for femoropopliteal in-stent occlusions in patients with symptomatic PAD. The main finding was that Auryon-assisted recanalization was associated with a significantly higher primary-strategy technical success rate compared with conventional endovascular recanalization. This difference was observed despite a significantly greater in-stent occlusion length in the Auryon group. Although no statistically significant baseline clinical differences were detected, SMDs showed clinically relevant imbalances in smoking history, chronic kidney disease, one-vessel runoff, and in-stent occlusion length. Post hoc Firth penalized logistic regression sensitivity analyses showed a consistent direction of effect for technical success after separate adjustment for selected imbalance variables. However, these analyses were exploratory and should not be interpreted as definitive evidence of a causal treatment effect. Importantly, the lower primary-strategy technical success rate in the conventional group should not be interpreted as a low final recanalization rate for contemporary EVT. In fact, six of nine conventional-group primary-strategy failures were successfully recanalized after distal retrograde bailout puncture, resulting in a final assisted recanalization rate of 88.5%. Therefore, the observed difference primarily reflects the ability of the initial recanalization strategy to achieve intraluminal crossing without unplanned additional access, rather than an absolute inability of conventional EVT to achieve final vessel recanalization.

At 12 months, primary patency was numerically higher after Auryon-assisted treatment, whereas target-vessel re-occlusion and CD-TLR were numerically lower. However, these estimates were imprecise, and the study was underpowered to reliably detect moderate between-group differences in 12-month outcomes.

Femoropopliteal in-stent occlusions remain among the most complex patterns of infrainguinal endovascular failure. In contrast to de novo stenoses or short re-stenotic lesions, complete in-stent occlusions require durable restoration of a true intraluminal channel within the pre-existing stent scaffold. Conventional wire-and-catheter recanalization may fail because of dense neointimal hyperplasia, thrombotic organization, calcific burden, or inability to maintain the guidewire within the stent lumen. These mechanisms may explain the lower technical success observed in the control group.

Laser atherectomy may offer procedural advantages at two distinct stages of treatment. First, when conventional guidewire progression is unsuccessful, controlled laser activation against the proximal occlusive cap can create a pilot channel within the pre-existing stent lumen. Alternating short laser advances with guidewire progression may thereby facilitate stepwise intraluminal crossing while reducing the likelihood of passage outside the stent scaffold. Second, after complete wire traversal, laser atherectomy can modify and debulk neointimal, thrombotic, and calcific material, improving lesion preparation before adjunctive balloon angioplasty and potentially reducing the need for extensive bailout stenting. This dual crossing and vessel-preparation role may have contributed to the higher technical success observed in the Auryon group, although the retrospective design does not allow a definitive causal relationship to be established. Although systematic IVUS was not available, several procedural observations may help explain the potential role of laser-assisted recanalization in this lesion subset. In complete femoropopliteal in-stent occlusions, conventional wires may be deflected at a resistant proximal cap, particularly when compact neointimal, thrombotic, or calcific material occupies the stent lumen. In other cases, the guidewire may partially enter the occluded stent but fail to maintain stable intrastent tracking, or the support catheter may not advance despite apparent wire progression. In this setting, short and controlled laser activation at the proximal cap may create a small pilot channel, reducing wire deflection and facilitating coaxial advancement within the pre-existing stent scaffold. Once partial wire passage has been obtained, stepwise laser advancement over the wire may further enlarge the intrastent channel and improve support for subsequent balloon angioplasty. These mechanisms remain hypothetical in the absence of systematic IVUS or histopathologic assessment, but they are consistent with the procedural rationale for using laser assistance when conventional wire-and-catheter crossing is unsuccessful. Previous studies evaluating laser atherectomy in femoropopliteal in-stent restenosis suggested that laser debulking combined with adjunctive balloon angioplasty or DCB angioplasty may improve acute luminal gain and reduce recurrent restenosis compared with balloon-based treatment alone [11,12]. However, much of the available evidence concerns excimer laser atherectomy or mixed restenotic lesions rather than complete in-stent occlusions treated with a 355 nm solid-state laser platform.

The Auryon system uses a 355 nm wavelength and short pulse duration. Experimental studies have investigated the interaction of this platform with atherosclerotic and calcified tissue, suggesting the potential for plaque modification with limited thermal injury [13,14,15]. However, the translation of these mechanistic observations into clinically meaningful advantages remains incompletely defined. Early clinical studies, including the EX-PAD-03 trial and subsequent IVUS-based evaluations, reported favorable acute procedural results and a low incidence of severe angiographic vessel injury after Auryon laser atherectomy followed by adjunctive balloon angioplasty [16,17]. These data provide a rationale for further clinical evaluation, but they should not be interpreted as definitive evidence of superiority in all infrainguinal lesion subsets.

These findings were subsequently supported by additional clinical experience with the same 355 nm laser platform. The B-Laser Investigational Device Exemption (IDE) study demonstrated favorable safety and effectiveness at 6 months in patients with infrainguinal PAD [18], while later real-world single-center studies of the commercially available Auryon system reported encouraging procedural performance and sustained clinical outcomes up to 12 months [19,20]. However, these studies included heterogeneous infrainguinal lesion subsets, whereas the present analysis specifically focused on complete femoropopliteal in-stent occlusions and incorporated a conventional recanalization control group.

The present findings should also be interpreted in the context of previous studies on femoropopliteal in-stent restenosis (ISR). Tosaka et al. demonstrated that the angiographic pattern of ISR has important prognostic implications, with totally occluded ISR lesions showing the highest rates of recurrent restenosis and recurrent occlusion after repeat intervention. In that study, class III ISR was associated with markedly worse 2-year outcomes than focal or diffuse non-occlusive ISR, supporting the concept that complete in-stent occlusion represents a distinct and particularly challenging subset [24]. In the prospective PATENT registry, excimer laser atherectomy for femoropopliteal ISR achieved high acute procedural success, although approximately one third of lesions were total occlusions and the study did not include a conventional recanalization control arm [25]. The randomized EXCITE ISR trial subsequently showed that excimer laser atherectomy followed by balloon angioplasty improved procedural success and short-term freedom from TLR compared with balloon angioplasty alone in femoropopliteal ISR, but again included mixed ISR patterns rather than exclusively complete in-stent occlusions [26]. Similarly, the FAIR trial demonstrated the superiority of DCB angioplasty over plain balloon angioplasty for SFA ISR, but the proportion of total occlusions was limited and the study did not evaluate laser-assisted crossing [27]. More recently, a network meta-analysis of endovascular treatments for femoropopliteal ISR suggested favorable 12-month patency and TLR outcomes with combined excimer laser atherectomy and DCB angioplasty, while emphasizing the need for more focused comparative studies [28]. Compared with these previous investigations, the present study specifically focused on complete femoropopliteal in-stent occlusions and evaluated whether a 355 nm solid-state laser platform could improve primary-strategy intraluminal recanalization without unplanned additional access.

In the present study, no procedure-related complication occurred in the Auryon group, and no distal embolization was observed. This finding is clinically relevant because distal embolization represents a potential concern during debulking of occlusive or restenotic femoropopliteal lesions, particularly in patients with limited runoff. In our series, active aspiration was used in all Auryon-assisted procedures, and catheter diameter was selected according to the occluded stent diameter and the estimated reference vessel diameter. This pragmatic approach reflects real-world use of the 2.0 mm and 2.35 mm catheters for femoropopliteal in-stent disease.

The significantly higher primary-strategy technical success rate observed with Auryon represents the most robust finding of this study. Technical success is particularly important in femoropopliteal in-stent occlusions because failure of conventional guidewire crossing may lead to alternative access attempts, direct stent puncture, more complex reinterventions, surgical bypass, or persistence of symptoms. In the present series, the ability to use the laser catheter before complete guidewire traversal provided an additional intraluminal crossing option when standard wire-and-catheter progression was unsuccessful. This endpoint should be interpreted as a predefined primary-strategy technical success rather than as assisted final procedural success. This approach may have underestimated the overall rate of final vessel recanalization after bailout maneuvers but allowed a consistent comparison between Auryon-assisted and conventional recanalization strategies. Although long-term patency remains the ultimate clinical objective, achieving safe and effective recanalization is the necessary first step, especially in patients with rest pain or tissue loss.

The 12-month outcomes should be interpreted cautiously. In the conditional analysis restricted to technically successful procedures, primary patency was numerically higher in the Auryon group, whereas target-vessel re-occlusion and CD-TLR were numerically lower. However, the study was underpowered to demonstrate statistically significant differences. Primary patency incorporated freedom from re-occlusion, duplex-detected hemodynamically significant restenosis, and clinically driven reintervention, thereby providing a broader assessment of treatment durability than re-occlusion alone. This conditional analysis describes the durability of successfully established recanalization but does not capture the clinical impact of initial technical failure.

To address this limitation, an exploratory full-cohort strategy-level analysis was performed in which initial technical failures were classified as endpoint failures. Twelve-month durable technical success was significantly higher in the Auryon group than in the control group (83.3% vs. 50.0%), as was technical success with freedom from CD-TLR (88.9% vs. 50.0%). These composite analyses provide a more comprehensive estimate of the effectiveness of the initial treatment strategy because they incorporate both the ability to establish target-vessel patency and its subsequent maintenance. Nevertheless, these findings remain exploratory and are driven in part by the substantial difference in initial technical success. They should therefore not be interpreted as definitive evidence of superior long-term patency after Auryon-assisted recanalization.

The potential economic implications of laser-assisted recanalization also warrant consideration. Laser atherectomy systems involve additional equipment costs and may increase procedural complexity compared with conventional wire-and-catheter recanalization. Therefore, improved primary-strategy technical success alone may not be sufficient to justify widespread adoption unless it is accompanied by clinically meaningful reductions in failed recanalization, unplanned bailout access, repeat reinterventions, hospitalization, limb-related complications, or overall resource utilization. In the present study, no formal cost-effectiveness analysis was performed, and the limited sample size and 12-month follow-up preclude any conclusion regarding economic value. Although the higher primary-strategy technical success and exploratory strategy-level outcomes may support further evaluation, future multicenter prospective studies with longer follow-up should incorporate health-economic endpoints, including device costs, procedural duration, adjunctive material use, need for bailout maneuvers, reinterventions, hospital stay, and limb-related outcomes.

In the present study, postprocedural antithrombotic therapy was standardized across both treatment periods according to the VOYAGER PAD regimen; therefore, differences in antithrombotic strategy are unlikely to explain the observed between-group outcomes. Beyond device-related performance and procedural success, outcomes after femoropopliteal revascularization also depend on appropriate secondary prevention of cardiovascular and limb events. Dual-pathway inhibition with low-dose rivaroxaban and aspirin has demonstrated clinical benefit in patients with stable PAD and following lower-extremity revascularization [22,23]. Nevertheless, the translation of randomized evidence into routine vascular practice remains heterogeneous. The RIVAS worldwide survey explored contemporary prescribing patterns and clinical perceptions regarding rivaroxaban among vascular surgeons, emphasizing the relevance of individualized antithrombotic decision-making after vascular interventions [29].

The present experience adds to the available literature by focusing specifically on femoropopliteal in-stent occlusions, rather than mixed infrainguinal lesion subsets. This distinction is relevant because in-stent occlusion poses different technical and biological challenges compared with de novo stenosis, chronic total occlusion, or non-occlusive in-stent restenosis. The study also provides a real-world comparison with conventional recanalization, which remains the most widely available treatment approach in many centers.

### Limitations

This study has several limitations. First, it was retrospective and non-randomized, with inherent risks of selection bias, information bias, and unmeasured confounding. Second, the control group was historical, introducing a potential era effect related to changes in operator experience, endovascular devices, medical therapy, and procedural strategies. The main intentional change between the two study periods was the availability and subsequent routine implementation of Auryon-assisted recanalization for femoropopliteal in-stent occlusions. The same operator team, treatment criteria, adjunctive lesion-treatment protocol after successful recanalization, antithrombotic regimen, and follow-up schedule were maintained across both periods. However, unmeasured real-world changes in practice, including incremental operator experience, guidewire and support-catheter selection, DCB platforms, and COVID-era effects on patient selection or follow-up intensity, cannot be fully excluded and may have influenced the comparison. Consequently, the observed difference in primary-strategy technical success should be interpreted as an association with the Auryon-era treatment strategy rather than as definitive proof of an isolated device-specific causal effect. Third, the sample size was limited, and the study was not powered to detect statistically significant differences in 12-month primary patency, target-vessel re-occlusion, CD-TLR, major amputation, or mortality. Although SMDs and post hoc Firth penalized logistic regression were used to assess baseline imbalance and the directional robustness of the primary-strategy technical-success finding, these analyses were limited by the small sample size, sparse number of failures, wide confidence intervals, and residual confounding. Propensity-score matching and inverse-probability weighting were considered but not performed because the limited cohort size and small number of outcome events would have produced an unstable analysis with substantial loss of sample size or imprecise weights. Therefore, residual confounding from measured and unmeasured baseline imbalance remains an unresolved limitation, and the Firth models should be regarded as hypothesis-generating sensitivity analyses rather than valid multivariable adjustment. Fourth, primary-strategy technical success was defined using a pragmatic, predefined, strategy-based angiographic criterion. Procedures requiring unplanned retrograde access or direct stent puncture were classified as failures of the predefined primary strategy, even if final vessel recanalization was obtained. This definition underestimated assisted final procedural success, particularly in the conventional group, in which six of nine primary-strategy failures were subsequently recanalized after distal retrograde bailout puncture. However, this approach preserved consistency across the study cohort and allowed comparison of the predefined recanalization strategies. Fifth, the prespecified primary endpoint was conditional on primary-strategy technical success, because primary patency could only be assessed after a patent treated segment had been established. Since technical failure was more frequent in the historical control group, this conditional analysis may have introduced post-treatment selection bias and should not be interpreted as a full-cohort estimate of treatment-strategy effectiveness. Exploratory full-cohort strategy-level composite analyses were performed to address this issue, but these endpoints were not prespecified and were strongly influenced by the between-group difference in initial technical success. Sixth, several granular procedural and anatomical variables were not systematically available, including laser fluence settings, pulse frequency, number of laser passes, fluoroscopy time, radiation dose, contrast volume, detailed crossing time, stent type, total stented length, time from initial stent implantation to occlusion, and previous treatments for in-stent restenosis. Although all included lesions were complete femoropopliteal in-stent occlusions and were therefore broadly consistent with Tosaka class III disease, formal adjudicated Tosaka classification and detailed morphological characterization of the occlusions were not systematically available. In particular, the distribution and length of neointimal, thrombotic, calcific, or neoatherosclerotic components could not be reliably assessed. Standardized assessment of stent deformation or fracture, IVUS findings, and formal runoff scoring was also not consistently performed. These limitations precluded a more detailed analysis of the mechanisms of in-stent occlusion and of lesion-level predictors of crossing success. Seventh, duplex ultrasound was the primary follow-up imaging modality, and routine computed tomography angiography was not performed in all patients at 12 months. Therefore, anatomical characterization of restenosis and re-occlusion mechanisms may have been limited. Future prospective studies should incorporate standardized post-treatment imaging, including computed tomography angiography and IVUS when feasible, to better characterize restenosis and re-occlusion mechanisms and identify lesion phenotypes most likely to benefit from laser-assisted recanalization.

Finally, follow-up was limited to 12 months. Longer follow-up is required to determine whether the higher technical success and strategy-level effectiveness observed with Auryon-assisted recanalization translate into improved long-term durability, reduced reintervention, and better limb-related outcomes.

## 5. Conclusions

In this retrospective comparative study, Auryon 355 nm laser atherectomy was associated with significantly higher primary-strategy technical success than conventional endovascular recanalization for femoropopliteal in-stent occlusions. This finding should be interpreted as an advantage of the predefined initial recanalization strategy, rather than as evidence of inferior final assisted recanalization with conventional EVT, which remained high after bailout maneuvers. The ability to use the laser both to facilitate intraluminal crossing and to modify intrastent occlusive material may represent a procedural advantage in complex lesions. In an exploratory full-cohort strategy-level analysis, 12-month durable technical success was also higher after Auryon-assisted treatment. Among technically successful procedures, primary patency was numerically higher, whereas target-vessel re-occlusion and CD-TLR were numerically lower in the Auryon group; however, these differences were not statistically significant.

## Figures and Tables

**Table 1:** Inclusion and exclusion criteria.

Inclusion Criteria	Exclusion Criteria
Rutherford category 3, 4, or 5	Rutherford category 0, 1, 2, or 6
Femoropopliteal in-stent occlusion >40 mm	Total occlusion length of the target femoropopliteal lesion >25 cm
At least one patent infragenicular vessel to the foot	Poor distal runoff, defined as partial or complete occlusion of all three tibial vessels
Estimated life expectancy >1 year	Multilevel disease requiring additional revascularization
	Pre-planned major amputation

**Table 2:** Baseline demographic and clinical characteristics of the overall study population and according to treatment group (*SMD: Standardized mean difference*).

Variable	Total N = 44	Auryon n = 18	Control n = 26	*p*-Value	SMD
Age, years	67.8 ± 9.3	68.5 ± 11.1	67.3 ± 9.1	0.695	0.12
Male sex	30 (68.1%)	11 (61.1%)	19 (73.1%)	0.611	0.26
BMI, kg/m^2^	26.8 ± 3.7	26.7 ± 2.5	26.9 ± 4.3	0.790	0.06
Smoking history	35 (79.5%)	17 (94.4%)	18 (69.2%)	0.097	0.69
Hypertension	36 (81.8%)	14 (77.7%)	22 (84.6%)	0.856	0.18
Diabetes mellitus	26 (59.1%)	12 (66.6%)	14 (53.8%)	0.590	0.26
Insulin-dependent diabetes	10 (22.7%)	5 (27.7%)	5 (19.2%)	0.764	0.20
Dyslipidemia	27 (61.3%)	11 (61.1%)	16 (61.5%)	1.000	0.01
Coronary artery disease	16 (36.3%)	8 (44.4%)	8 (30.7%)	0.542	0.29
Previous myocardial infarction	4 (9.1%)	1 (5.5%)	3 (11.5%)	0.884	0.22
Chronic kidney disease	4 (9.1%)	0 (0%)	4 (19.2%)	0.135	0.60
Previous amputation	2 (4.5%)	0 (0%)	2 (7.6%)	0.639	0.41
Rutherford category 3	22 (50.0%)	8 (44.4%)	14 (53.8%)	0.759	0.19
Rutherford category 4	17 (38.6%)	8 (44.4%)	9 (34.6%)	0.731	0.20
Rutherford category 5	5 (11.4%)	2 (11.1%)	3 (11.5%)	1.000	0.01

**Table 3:** Anatomical characteristics of the femoropopliteal in-stent occlusions and procedural variables according to treatment group (*SMD: Standardized mean difference*).

Variable	Total N = 44	Auryon n = 18	Control n = 26	*p*-Value	SMD
Popliteal involvement	13 (29.5%)	4 (22.2%)	9 (34.6%)	0.582	0.28
Reference vessel diameter, mm	5.2 ± 0.6	5.2 ± 0.5	5.1 ± 0.6	0.497	0.18
Total occlusion length, mm	144 ± 50.3	137.2 ± 41.7	150 ± 55.7	0.389	0.26
In-stent occlusion length, mm	86.8 ± 24.1	97.2 ± 26.9	79.6 ± 19.4	0.024	0.75
One patent runoff vessel	16 (36.3%)	10 (55.5%)	6 (23.0%)	0.059	0.71
Two patent runoff vessels	11 (25.0%)	2 (11.1%)	9 (34.6%)	0.156	0.58
Three patent runoff vessels	17 (38.6%)	6 (33.3%)	11 (42.3%)	0.774	0.19
Contralateral femoral access	6 (13.6%)	2 (11.1%)	4 (15.3%)	1.000	0.13
Introducer diameter, Fr	6.2 ± 0.6	6.7 ± 0.4	5.8 ± 0.5	<0.001	1.99
Balloon diameter, mm	5.0 ± 0.4	5.1 ± 0.3	4.9 ± 0.5	0.143	0.49
Balloon length, mm	123.6 ± 32.8	125.5 ± 26.8	122.3 ± 36.9	0.737	0.10
Balloon pressure, atm	11.7 ± 1.4	11.5 ± 1.2	11.9 ± 1.5	0.397	0.29
Number of balloons per patient	1.1 ± 0.3	1.1 ± 0.3	1.1 ± 0.2	0.686	0.00
Stent relining	4 (9.1%)	1 (5.5%)	3 (11.5%)	0.884	0.22

**Table 4:** Procedural and 12-month outcomes (*The p-value for primary patency was calculated using the log-rank test; p-values for the remaining categorical endpoints were calculated using Fisher’s exact test; Absolute differences were calculated as Auryon minus control*).

Endpoint	Population	Total	Auryon	Control	Absolute Difference, % (95% CI)	*p*-Value
Primary strategy technical success	Full cohort	34/44 (77.3%)	17/18 (94.4%)	17/26 (65.4%)	29.1 (3.8 to 48.8)	0.031
Final assisted recanalization after bailout	Full cohort	40/44 (90.9%)	17/18 (94.4%)	23/26 (88.5%)	6.0 (−15.6 to 24.0)	0.634
Durable technical success at 12 months	Full cohort	28/44 (63.6%)	15/18 (83.3%)	13/26 (50.0%)	33.3 (4.5 to 54.3)	0.030
Primary-strategy technical success with freedom from CD-TLR	Full cohort	29/44 (65.9%)	16/18 (88.9%)	13/26 (50.0%)	38.9 (10.7 to 58.5)	0.010
Primary patency at 12 months	Technical successes	28/34 (82.4%)	15/17 (88.2%)	13/17 (76.5%)	11.8 (−14.8 to 37.0)	0.37
CD-TLR	Technical successes	5/34 (14.7%)	1/17 (5.9%)	4/17 (23.5%)	−17.6 (−41.9 to 7.7)	0.335
Major amputation	Full cohort	3/44 (6.8%)	1/18 (5.6%)	2/26 (7.7%)	−2.1 (−19.2 to 18.8)	1.000
All-cause mortality	Full cohort	1/44 (2.3%)	0/18 (0%)	1/26 (3.8%)	−3.8 (−18.9 to 14.0)	1.000
Procedure-related complications	Full cohort	1/44 (2.3%)	0/18 (0%)	1/26 (3.8%)	−3.8 (−18.9 to 14.0)	1.000

**Table 5:** Procedural time and length of hospital stay among technically successful procedures (*Data are presented as mean ± standard deviation. Mean differences were calculated as Auryon minus control*).

Variable	Total, N = 34	Auryon, n = 17	Control, n = 17	Mean Difference	95% CI	*p*-Value
Procedural time, min	84.1 ± 36.9	96.9 ± 45.1	75.2 ± 27.5	21.7	−2.83 to 46.2	0.080
Length of hospital stay, days	2.0 ± 2.1	2.0 ± 1.8	2.1 ± 2.4	−0.1	−1.40 to 1.17	0.857

## Data Availability

The data presented in this study are available from the corresponding author upon reasonable request, subject to privacy and institutional restrictions.

## Supplementary Materials

The following supporting information can be downloaded at: https://www.mdpi.com/article/10.3390/biomedicines14071538/s1.

## Abbreviations

The following abbreviations are used in this manuscript:

BMI	Body mass index
CD-TLR	Clinically driven target-lesion revascularization
CI	Confidence interval
CLTI	Chronic limb-threatening ischemia
DCB	Drug-Coated Balloon
IDE	Investigational Device Exemption
ISR	In-Stent restenosis
IVUS	Intravascular ultrasound
PAD	Peripheral arterial disease
PLAN	Patient risk estimation, Limb staging, and ANatomic pattern of disease
PSVR	Peak systolic velocity ratio
SMD	Standardized mean difference
