# Supplementary material for: Auryon 355 nm Laser Atherectomy for Femoropopliteal In-Stent Occlusions: A Retrospective Comparative Study of Technical Success and 12-Month Outcomes
Source: Biomedicines. 2026 Jul 9;14(7):1538. doi: 10.3390/biomedicines14071538 (PMC13404598; doi:10.3390/biomedicines14071538)
Supplement: Supplementary file 1 [file biomedicines-14-01538-s001.zip › biomedicines-4409025-supplementary.pdf]

Supplementary Table S1. Case-by-case characterization of primary-strategy technical failures.

Study ID	Treatment strategy	Rutherford category	Target vessel	RVD (mm)	Total occlusion length (mm)	In-stent occlusion length (mm)	Patent BTK runoff vessels	Primary access	Distal retrograde bailout outcome	Failure classification
5	Conventional	4	SFA	5	150	80	2	Contralateral crossover femoral	Successful	Primary-strategy failure; final assisted recanalization achieved after distal retrograde bailout puncture
7	Conventional	3	SFA	5	100	60	1	Contralateral crossover femoral	Successful	Primary-strategy failure; final assisted recanalization achieved after distal retrograde bailout puncture
10	Conventional	5	SFA	4	190	100	1	Contralateral crossover femoral	Unsuccessful	Absolute final technical failure despite distal retrograde bailout puncture
12	Conventional	4	SFA	5	200	80	2	Contralateral crossover femoral	Unsuccessful	Absolute final technical failure despite distal retrograde bailout puncture
16	Conventional	4	SFA	5	80	60	3	Ipsilateral antegrade femoral	Successful	Primary-strategy failure; final assisted recanalization achieved after distal retrograde bailout puncture
19	Conventional	4	SFA + popliteal artery	4	200	100	2	Contralateral crossover femoral	Unsuccessful	Absolute final technical failure despite distal retrograde bailout puncture
20	Conventional	5	SFA	5	100	60	2	Ipsilateral antegrade femoral	Successful	Primary-strategy failure; final assisted recanalization achieved after distal retrograde bailout puncture
22	Conventional	5	SFA	6	80	60	1	Contralateral crossover femoral	Successful	Primary-strategy failure; final assisted recanalization achieved after distal retrograde bailout puncture
26	Conventional	3	SFA	6	160	100	3	Contralateral crossover femoral	Successful	Primary-strategy failure; final assisted recanalization achieved after distal retrograde bailout puncture
41	Auryon-assisted	3	SFA	5	80	60	3	Contralateral crossover femoral	Unsuccessful	Absolute final technical failure despite combined guidewire-/laser-assisted crossing and distal retrograde bailout puncture

Before classification as primary-strategy failure, all conventional-group failure cases underwent standard crossing attempts, including guidewire escalation, support-catheter optimization, and selective knuckle-wire technique when intrastent progression could be maintained. These maneuvers are not repeated row by row in the table. Distal retrograde bailout puncture was then attempted in all primary-strategy failure cases. *(BTK, below-the-knee; RVD, reference vessel diameter; SFA, superficial femoral artery.*)

Supplementary Table S2. Post hoc Firth penalized logistic regression sensitivity analysis for technical success.

Model	Adjustment variable	OR for Auryon vs. control	95% CI
Unadjusted	No adjustment	9.00	1.02-79.4
Firth model 1	In-stent occlusion length	10.8	1.05-132.0
Firth model 2	One-vessel runoff	11.6	1.03-155.0
Firth model 3	Smoking history	8.4	0.83-116.0

Supplementary Table S3. Post hoc power-context analysis for non-significant 12-month outcomes.

Endpoint	Analysis population	Observed Auryon rate	Observed control rate	Observed absolute difference	Power-context interpretation
**Primary patency at 12 months**	17 vs. 17	88.2%	76.5%	+11.8 pp	80% power not attainable within feasible 0–100% bounds using observed control rate; even 100% vs. 76.5% would provide only approximately 63% power
**Target-vessel re-occlusion**	17 vs. 17	11.8%	23.5%	−11.8 pp	80% power not attainable; even 0% vs. 23.5% would provide only approximately 63% power
**CD-TLR**	17 vs. 17	5.9%	23.5%	−17.6 pp	80% power not attainable; even 0% vs. 23.5% would provide only approximately 63% power
**Major amputation**	18 vs. 26	5.6%	7.7%	−2.1 pp	Event rate too low for reliable between-group inference
**All-cause mortality**	18 vs. 26	0%	3.8%	−3.8 pp	Event rate too low for reliable between-group inference
**Procedure-related complications**	18 vs. 26	0%	3.8%	−3.8 pp	Event rate too low for reliable between-group inference

Power-context estimates were calculated post hoc using the available analysis population, two-sided α = .05, and 80% power. These estimates are descriptive and were used only to contextualize the imprecision of non-significant secondary outcomes. CD-TLR, clinically driven target-lesion revascularization; pp, percentage point
